# Potential anthelmintic effect of chitosan on *Syphacia muris* infecting Wistar rats: biochemical, immunological, and histopathological studies

**DOI:** 10.1038/s41598-024-52309-8

**Published:** 2024-02-03

**Authors:** Nesma A. Mostafa, Salwa A. H. Hamdi, Mona F. Fol

**Affiliations:** Zoology Department, Faculty of Science, Cairo University, Giza, Egypt

**Keywords:** Biological techniques, Biotechnology, Immunology, Zoology

## Abstract

Natural products extracted from animal sources have many biological activities, such as chitosan, which is being researched for its medicinal or therapeutic potential. *Syphacia muris* is the most well-known intestinal nematode, infecting laboratory rats and influencing their immune systems. In this study, we looked at the anthelminthic activity of chitosan particles against *S. muris* infection using biochemical, immunological, and histopathological methods. Chitosan particles were characterized using Fourier-transform infrared spectroscopy (FTIR). Rats were separated into four groups, each consisting of seven individuals (n = 7). The first group was the control (non-infected), the second group was infected, and both groups received 0.5 ml of 1% glacial acetic acid orally. The third group was the infected group (treated), and the fourth group (normal) received 0.5 ml of 30 mg/kg/day chitosan dissolved in 1% glacial acetic acid for 14 days using gavage. Liver and kidney parameters, oxidative stress markers, serum levels of cytokines (IFN-γ, IL-5, IL-13, IL-33, and IL-10), as well as immunoglobulins (total IgE and IgG), were assessed. Histological examinations of host tissues (intestine, liver, kidney, and spleen) were also performed. Following chitosan treatment, a significant decrease in worm count (*P* < 0.05) was indicated; this was associated with an enhancement of biochemical and oxidative stress biomarkers, which were altered due to infection. Moreover, immunological analysis revealed a significant drop in INF-γ, IL-5, IL-13, and IL-33 levels and total immunoglobulins (IgE and IgG) as well as an improvement in rat tissues. Conclusively, this study showed the anthelminthic effect of chitosan against *S. muris* infection.

## Introduction

*Syphacia muris* is the most prevalent pinworm in laboratory rats, and even under well-managed conditions, it is extremely difficult to keep them free of infection^1^. In addition to its zoonotic ability, they can affect the outcome findings^2,3^. For preventative and curative purposes, many gastrointestinal helminths have been treated with traditional anthelmintic drugs that are currently available^4^. Due to health risks associated with this chemotherapy to reduce helminth infection^5^, natural products are increasingly being promoted as alternatives to improve various aspects of human and animal health^6,7^. Bioactive compounds of natural products found in aquatic organisms^8,9^, considered eco-friendly used in medical, agricultural, food, and environmental industries due to their especially renewable, sustainable, and nontoxic properties^10^. Polysaccharide polymers are the most efficient applicants of biomedical products^11^. Chitosan an amino polysaccharide created by deacetylating the chitin in crustacean shells, typically from shrimp and crustaceans processing wastes^12^, has received much interest as an immunostimulant due to some of its characteristics like anti-inflammatory, antioxidant, anticancer and antimicrobial properties^13^.

Recent studies stated that chitosan has been selected as an effective drug for delivering chemotherapeutic agents due to its distinctive characteristics, which include a sustained circulation capacity, high drug loading capacity, multifunctionality, efficient drug release at cancerous sites, elimination of cytotoxicity towards non-cancerous cells, appropriate targeting, and cell membrane permeability facilitated by the chemical structure's primary amine group^14–16^. Furthermore, it has been demonstrated that chitosan given orally decreases serum concentrations of the pro-inflammatory cytokines TNF-α and IL-6. These cytokines are responsible for inducing leukocyte activation and tissue accumulation, and they play a substantial role in inflammatory disorders^17^. Therefore, this study aimed to investigate the anthelminthic activity of chitosan particles against *Syphacia muris* naturally infected Wistar rats.

## Material and methods

### Chitosan preparation

*Procambarus clarkii* (Crustacea: Cambaridae) were collected from the River Nile at Giza Governorate, Egypt. They were placed in plastic bags and transferred to Cairo University Laboratory of Invertebrates, Zoology Department, Faculty of Science. Shells were dissected and scraped to remove tissue before being rinsed and dried in an oven for 6 h at 60 °C. The product is homogenized using an electric blender to get crawfish powder that passes through 300μ sieve^18^. Using the methods stated by Hadi^19^, four different procedures; deproteinization, demineralization, decolorization, and deacetylation were performed to generate chitosan particles from the powder.

### Characterization of chitosan

#### Fourier-transform infrared spectroscopy (FTIR)

The extracted chitosan was rigorously mixed with potassium bromide then the dry material pressed to produce a homogenous sample/KBr disk. A Perkins-Elmer spectrometer (Spectrum RX I, MA, USA) was used to measure the infrared spectra between 3434.6 and 420.406 cm^−1^ with a tablet containing KBr and chitosan at a resolution of 4 cm^−1^.

#### Experimental design

Twenty-eight male albino Wistar rats weighing 200–240 g were purchased from the National Organization for Drug Control and Research, then transferred to the Laboratory of Invertebrates, Zoology Department, Faculty of Science, Cairo University. The procedures were approved by Cairo University Institutional Animal Care and Use Committee (CU-IACUC), and all methods were performed in accordance with the relevant guidelines and regulations. Experimental animals were kept at room temperature with a 12 h light/dark cycle and fed standard food and water ad libitum. According to Meade and Watson^20^, the natural infection with *S. muris* was detected in the feces of the investigated rats after acclimatization for 7 days using perianal cellophane tape. Rats were separated into four groups, each consisting of seven individuals (n = 7). The first group was the control (non-infected), the second group was infected, and both groups received 0.5 ml of 1% glacial acetic acid orally. The third group was the infected group (treated), and the fourth group (normal) received 0.5 ml of 30 mg/kg/day chitosan dissolved in 1% glacial acetic acid for 14 days using gavage^21^. At the end of the experiment, all rats were anesthetized by intraperitoneal injection of 10% pentobarbital sodium solution (3 ml/kg). The stomach, small intestine, caecum, and colon were separated from the surrounding tissue and placed into physiological saline. They were opened longitudinally and examined for helminth parasites; then, helminths were carefully removed, identified, and counted under a stereoscopic microscope.

#### Microscopic examination

For light microscopic examination, parasites were cleared with lactophenol. The recovered nematodes were photographed by A LEICA DM 750 microscope supplied with a LEICA ICC 50 HD camera and identified based on key suggested by Pinto et al.^22^. Worms were fixed for scanning electron microscopy in a solution of 3% glutaraldehyde, washed in 0.1 M sodium cacodylate buffer (pH 7.4), dehydrated through a graded ethanol series, and dried at 30 °C for 30 min using a critical point drier (LEICA, EM CPD300). Dried specimens were mounted on SEM stubs, coated with gold, and examined with a JEOL JSM-5200 SEM (Tokyo, Japan) using an accelerating voltage 25 kV. All body measurements are presented as means in mm ± SEM. According to Bush et al.^23^, mean intensity was calculated, and the percent of reduction of the recovered worms was determined.

$$Mean\, intensity=\frac{\mathrm{Total\, number\, of\, parasites }}{\mathrm{Number\, of\, infected\, hosts}}$$

$$Percent\, of\, reduction=\frac{Nt-Nc }{ Nc} \times 100$$

where N_c_: is the number of viable parasites before treatment. N_t_: is the number of viable parasites after treatment.

### Assessment of biochemical parameters

#### Tissue preparation

Kidney and liver tissues were weighed and homogenized (10% wt/vol) in an ice-cold (0.1 M Tris HCl buffer, pH 7.4) using a glass Dounce homogenizer (Swedesboro, USA). The homogenates were centrifuged for 15 min at 3000 rpm at 4 °C, and the obtained supernatant was kept at − 80 °C.

#### Kidney and liver function tests

The collected supernatants were utilized to estimate renal function markers, creatinine, and uric acid, as well as liver markers, alkaline phosphatase (ALP), aspartate aminotransferase (AST), and alanine aminotransferase (ALT) using Bio Diagnostic kits, Egypt.

#### Oxidative stress markers

The supernatant of liver homogenate was used to determine lipid peroxidation via malondialdehyde (MDA) formation according to Buege and Aust^24^. The stress-induced production of nitric oxide (NO) was measured using the method described by Montgomery and Dymock^25^. Also, glutathione reduced (GSH), the primary thiol was determined by the procedure of Beutler et al.^26^. Catalase (CAT), and superoxide dismutase (SOD) activities were estimated^27,28^ using Bio Diagnostic kits in Egypt.

#### Determination of cytokines and antibodies levels

Blood samples were collected then were centrifuged at 3000 rpm for 20 min. The clear non-hemolyzed sera were kept at − 80 °C. Levels of interleukins (IFN-γ, IL-5, and IL-10, IL-13, IL-33) and antibodies (Total IgE, IgG) were quantified by ELISA according to the manufacturer’s instructions (SUNLONG Biotech Co., Inc., China). All optical densities were obtained at 450 nm. Antibody and cytokine concentrations were expressed in pg/ml.

### Histopathological studies

Rat tissues (intestine, liver, kidney, and spleen) were removed, rinsed with saline, and fixed in 10% buffered formalin. Samples were dehydrated in ascending alcohol concentrations, cleared with xylene, embedded in paraffin, and stained with hematoxylin and eosin (H&E). The stained sections were investigated and photographed using a LEICA DM 750 light microscope with a LEICA ICC 50 HD camera.

### Statistical analysis

Data were presented as mean ± SEM and analyzed using one-way ANOVA in Statistical Processor Systems Support, SPSS software, version 20, followed by a Duncan post hoc test to measure the differences between the studied groups, and a t-test was used to determine the significant difference of the mean intensity between infected and treated groups. Values was considered statistically significant at (*P* < *0.05*).

### Ethics approval and informed consent

The current study was performed in accordance with ARRIVE guidelines. The animal experiments in this study were approved by the Cairo University Institutional Animal Care and Use Committee (CU-IACUC), under the relevant document (No. CU/I/F/75/19).

## Results

### Characterization of chitosan

**Figure 1:**
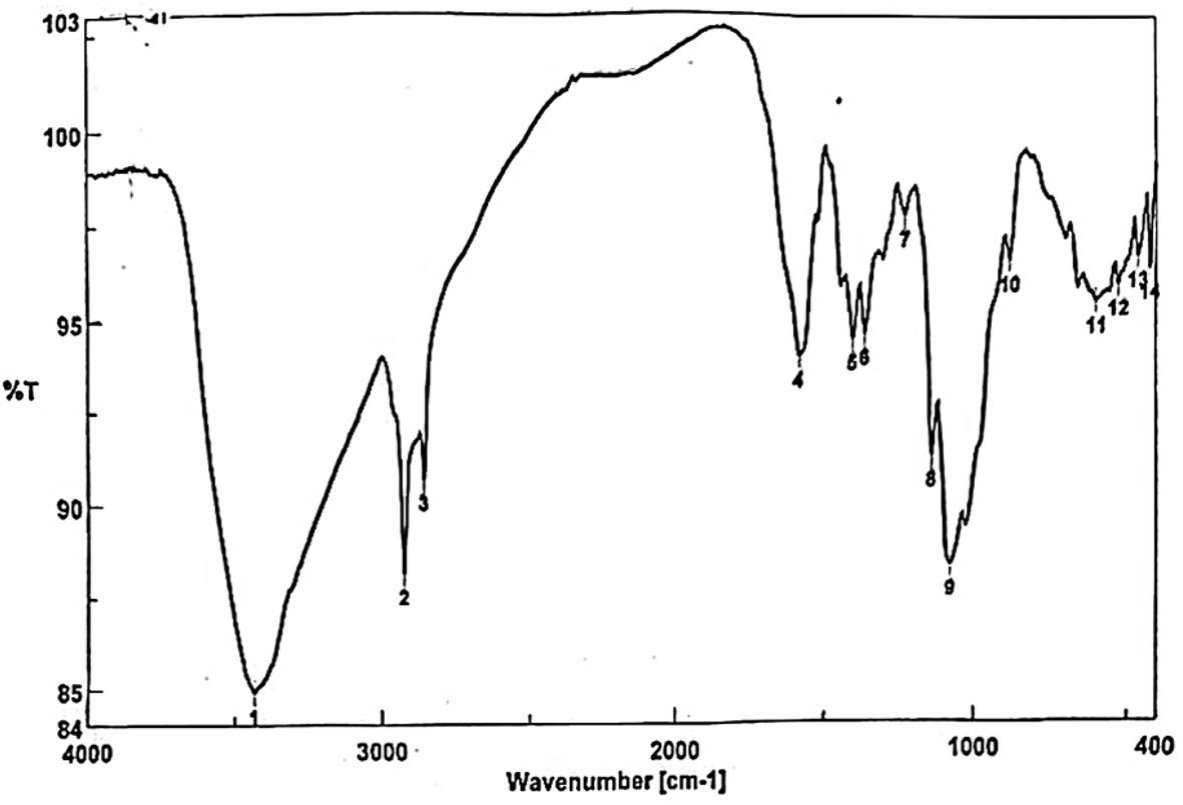
FTIR spectrum of chitosan with the characteristic signs as evidence.

FTIR spectroscopy was used to characterize chitosan particles, and the spectrum revealed peaks corresponding to their functional groups (Fig. 1 and Table 1). The NH stretching peaks proved the interference between the NH and OH groups. A strong band in the region 3434.6 corresponds to N–H and O–H stretching and intramolecular hydrogen bonds. The absorption bands at 2917.77 and 2851.24 cm^−1^ can be attributed to C-H symmetric and asymmetric stretching, respectively, these bands have polysaccharide properties. The bands at around 1595.81 cm^−1^ (C=O stretching of amide I) and 1250.61 cm^−1^ (C–N stretching of amide III) revealed the presence of residual N-acetyl groups. A band represents the N–H bending of the main amine at 1595.81 cm^−1^. The absorption band at 1153.22 cm^−1^results from the asymmetric stretching of the C–O–C bridge, and the bands at 1153.22 and 1080.91 cm^−1^ demonstrate C–O stretching. Commonly, chitosan, which is derived from animals, has a chance to be contaminated by glycosaminoglycans (GAGs). The signal at 1250.61 cm^−1^ is very small and, therefore, does not correspond to sulfate groups, thus ruling out contamination of chitosan by GAGs. This signal is assigned as the bending vibrations of hydroxyls in chitosan. The signal at 895.77 cm^−1^ corresponds to the C-H bending out of the plane of the ring of monosaccharides.

**Table 1:** FTIR peak spectra showing the functional groups of chitosan particles.

No	Band (cm^−1^)	Functional group
1	3434.6	Corresponds to N–H and O–H stretching
2	2917.77	C–H symmetric stretching
3	2851.24	C–H asymmetric stretching
4	1595.81	C=O stretching of amide I
5	1250.61	C–N stretching of amide III
6	1595.81	N–H bending
7	1153.22	C–O–C bridge
8	1153.22	C–O stretching
9	1080.91	C–O stretching
10	1250.61	Sulfate group
11	895.77	C–H bending

### Anthelmintic effects of chitosan

#### Worm burden

The number of worms per infected rat ranged from 4 to 12 and per treated one from 0.00 to 3. Following chitosan administration, the worm count was reduced by 87.5% (*P* < 0.05) in treated rats compared to untreated ones, and the mean intensity of infection was lowered from 8.00 ± 1.04 to 1.00 ± 0.43 in the treated group (Table 2).

**Table 2:** Mean intensity and percent of reduction of worm count after chitosan treatment.

Groups	Total worm count	Intensity of infection	Reduction percentage
Non-infected (control)	0.00	0.00	–
Infected	56	8.00 ± 1.04	–
Infected (treated)	7	1.00 ± 0.43*	87.5

Data represented as mean ± SEM (n = 7), asterisk represents the significance level (*P < *0.05*).

#### Microscopic examination (based on 5 mature specimens)

**Figure 2:**
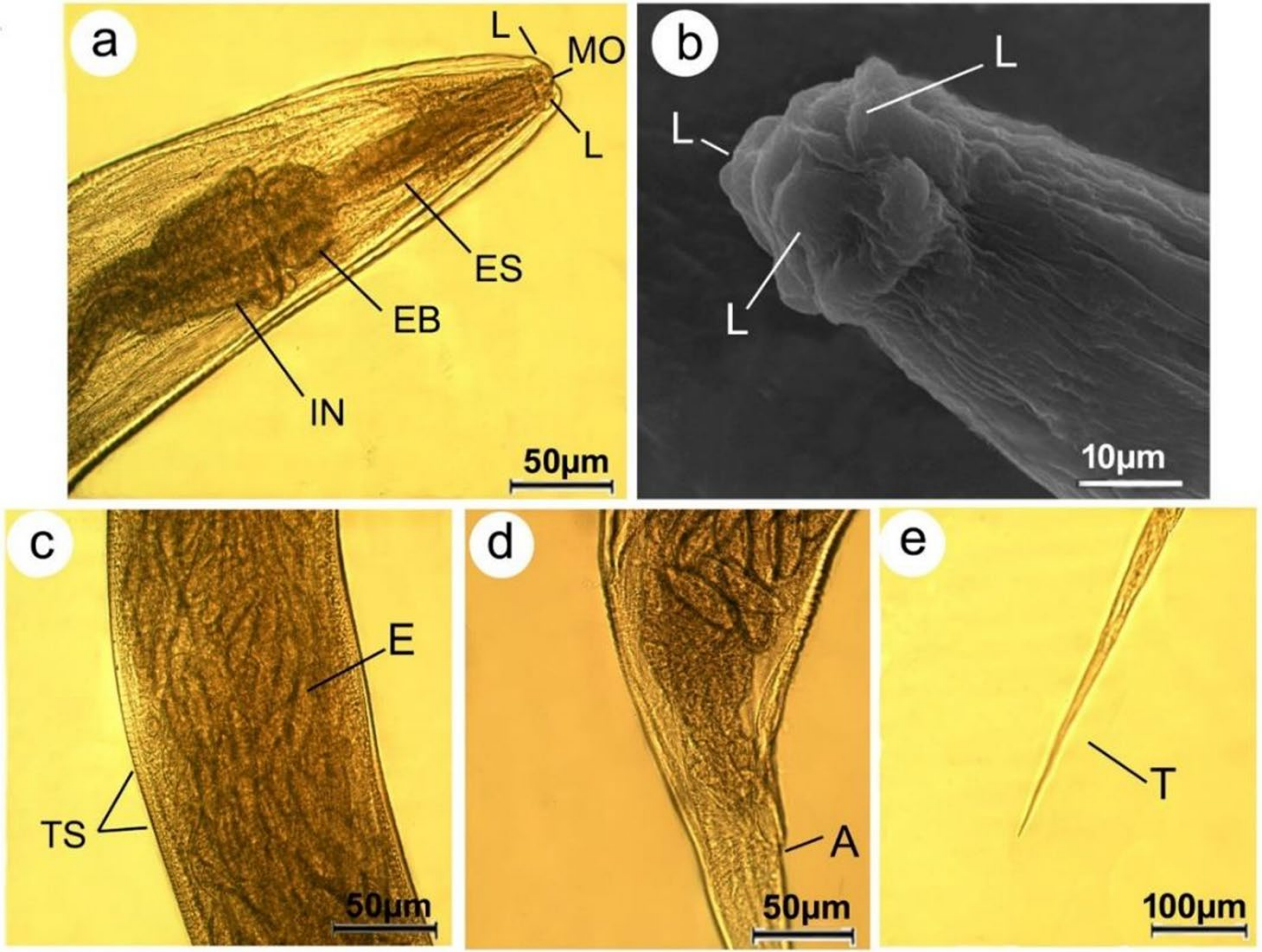
Photomicrographs of female *Syphacia muris* isolated from untreated rats cleared with lactophenol (**a**) anterior region showing mouth (MO), oesophagus (ES), oesophageal bulb (EB), intestine (I) (**b**) scanning electron micrograph showing head region supplied by three lips (L) (**c**) Mid-body region covered by cuticle with transverse striations (TS) and uterus filled with eggs (E) (**d**) posterior end showing anal opening (A) (**e**) high magnification of tail tip (T).

**Figure 3:**
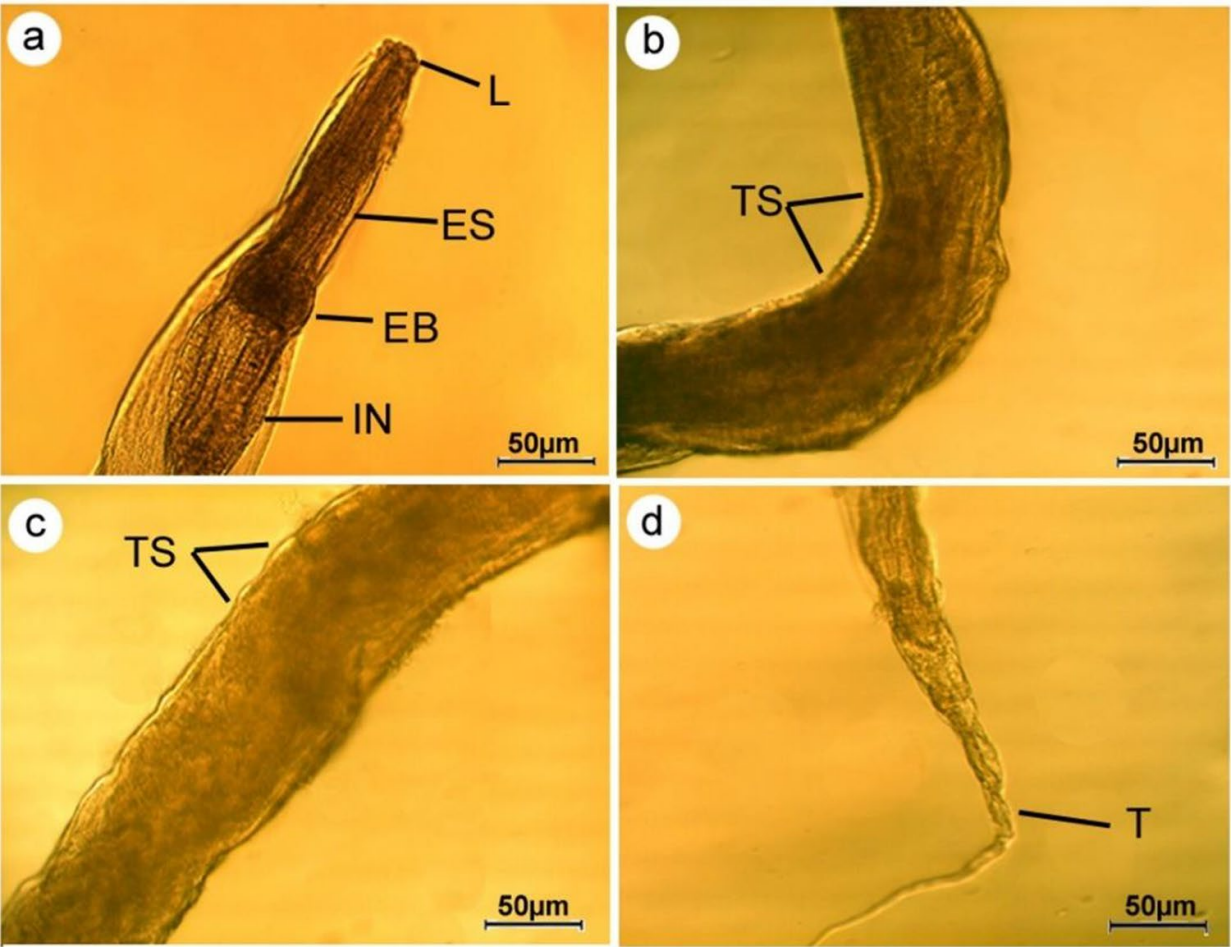
Photomicrographs of *Syphacia muris* isolated from treated rats displaying (**a**) cephalic region with indistinguishable lips (**b**) wrinkled body surfaces (**c**) cuticle striation becomes flexible with appearance of noticeable pits (**d**) deformation of tail region.

All the collected worms in this study were females *S. muris.* The bodies were between 2.8 and 5.3 mm in length and 0.14 and 0.16 mm in width, with creamy bodies and slender posterior extremities. The heads were bulbous with triradiate small mouth openings surrounded by three equal, well-developed fleshy lips (one dorsal and two ventrolateral) devoid of labial papillae (Fig. 2a,b). Buccal cavities led to esophagi, which were subdivided into anterior cylindrical and globular bulbs, and the latter led to simple tubular intestines via intestinal valves (Fig. 2a). The body cuticle was found to be transversally annulated. The uterus occupies nearly the bulk of the body and is densely packed with eggs (Fig. 2c), which were elliptical, compressed on one side, and measured 70 × 32 µm in length. Posterior extremity with an anal opening (Fig. 2d) and a pointed tail measured 0.58–0.67 mm long (Fig. 2e). On the other hand, the recovered worms showed morphological changes such as a wrinkled cephalic region with distorted lips after chitosan treatment (Fig. 3a). The cuticle was also disturbed resulting in visible pits on the body's surfaces (Fig. 3b,c). In addition, the tail area was completely deformed (Fig. 3d).

### Biochemical parameters

#### Liver and kidney biomarkers

**Figure 4:**
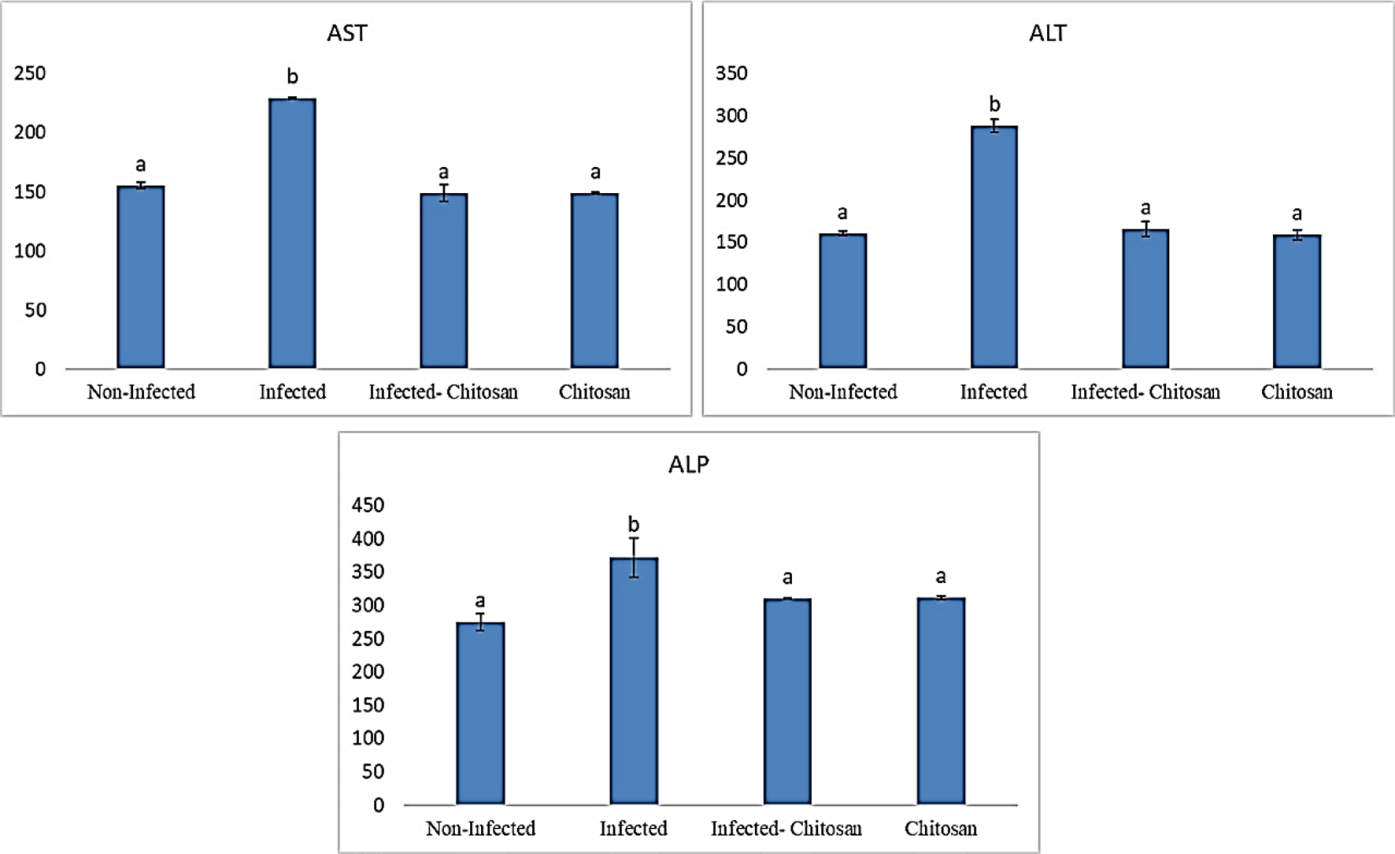
Enzymatic levels of aspartate transaminase (AST), alanine transaminase (ALT), and alkaline phosphatase (ALP) in all studied groups. Data represented as mean ± SEM (n = 7), values with different superscript letters are considered statistically significant (*P* < 0.05).

**Figure 5:**
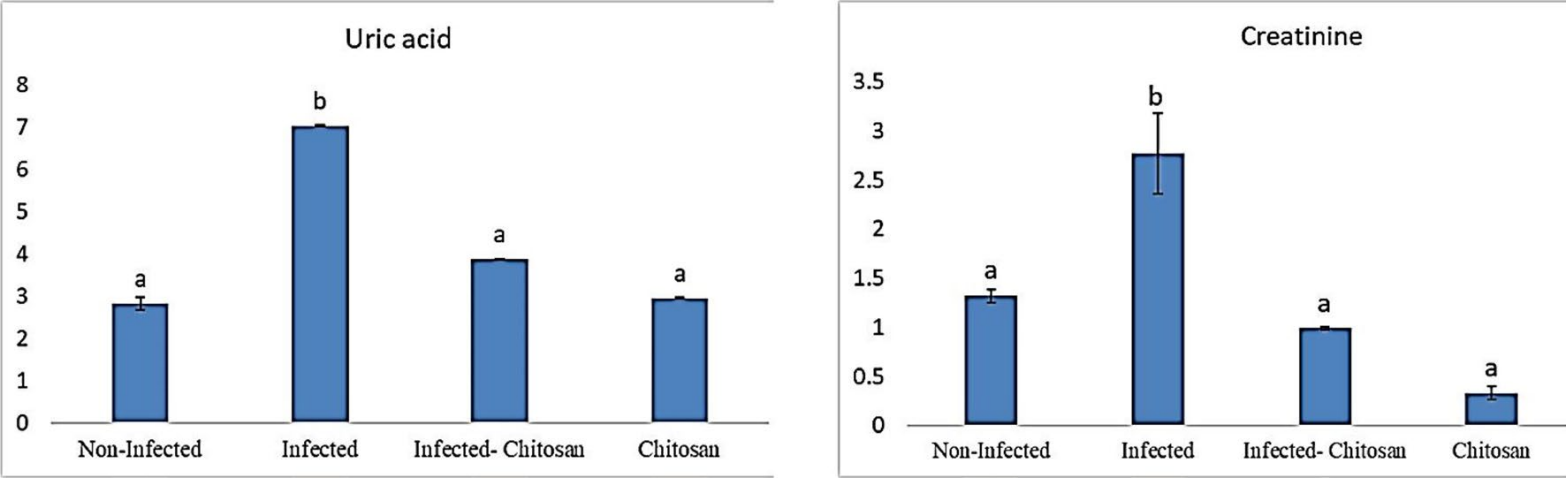
Levels of uric acid and creatinine in all studied groups. Data represented as mean ± SEM (n = 7), values with different superscript letters are considered statistically significant (*P* < 0.05).

Natural infection with *S. muris* caused biochemical changes in rat tissues, with substantially higher (*P* < 0.05) levels of aspartate aminotransferase (AST), alanine aminotransferase (ALT), and alkaline phosphatase (ALP) (Fig. 4), as well as creatinine, and uric acid (Fig. 5) compared to the control one. These levels appeared to have been recovered in chitosan-treated rats relative to the infected group.

#### Levels of oxidative stress markers

**Figure 6:**
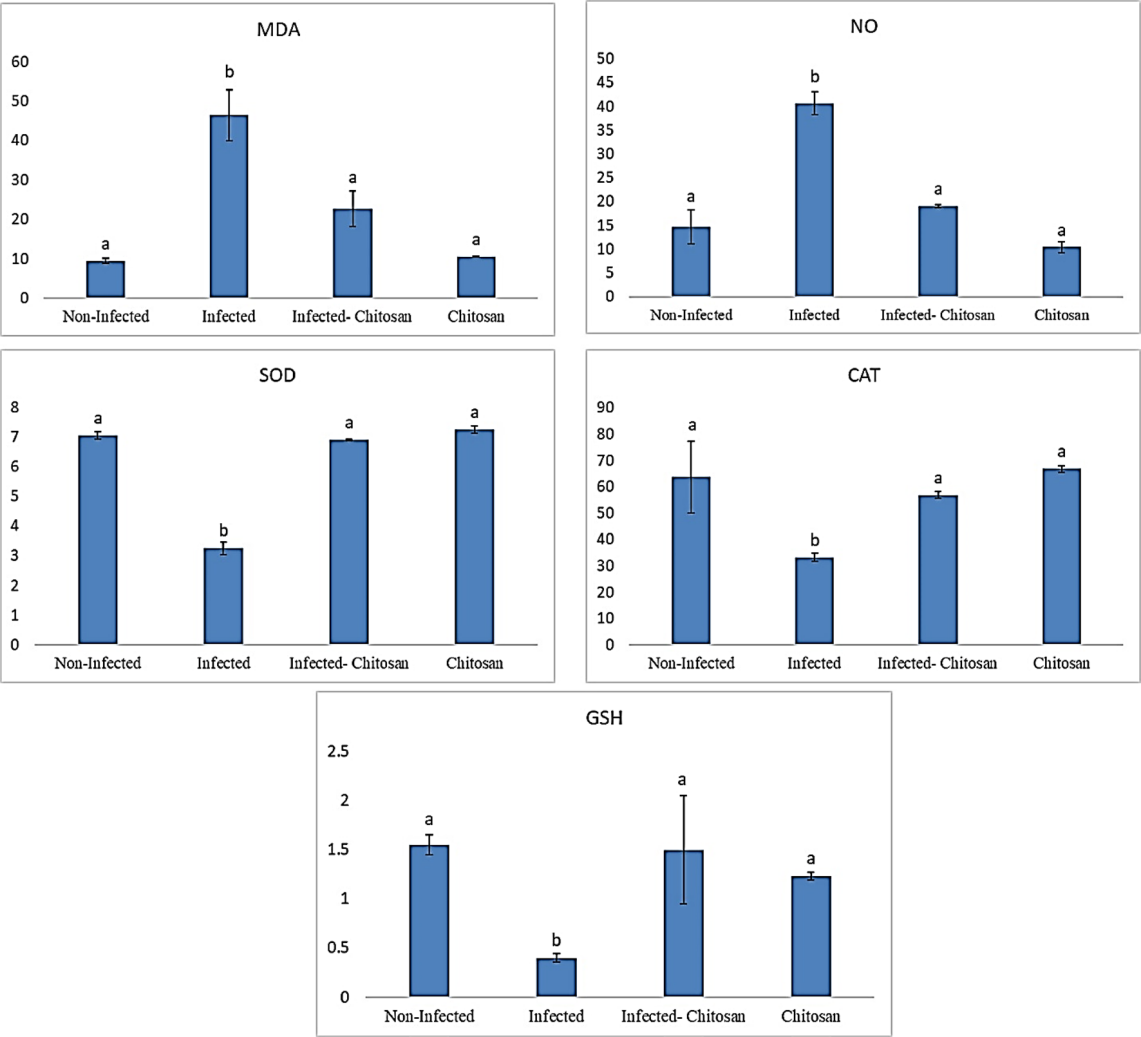
Levels of oxidative stress markers; malondialdehyde (MDA), nitric oxide (NO), superoxide dismutase (SOD), catalase (CAT) and reduced glutathione (GSH) in all studied groups. Data represented as mean ± SEM (n = 7), values with different superscript letters are considered statistically significant (*P* < 0.05).

As shown in Fig. 6, a significant (*P* < 0.05) increase in MDA and NO levels was observed in the infected group and a substantial drop (*P* < 0.05) in GSH content, CAT, and SOD activities was also recorded compared to the control group. In contrast, the antioxidant levels were returned to normal values following chitosan treatment.

### Immunological study

**Figure 7:**
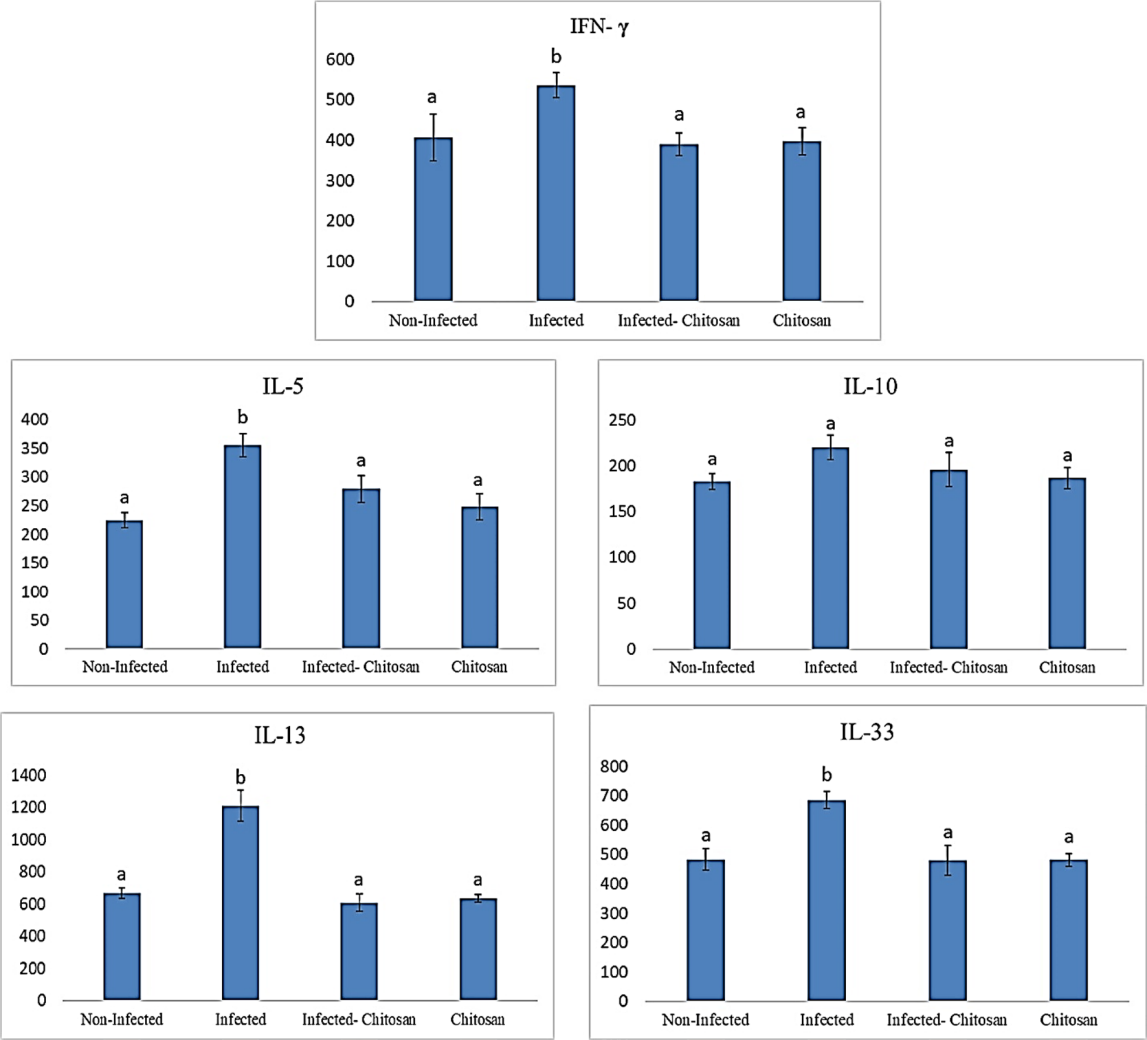
Cytokine levels of IFN-γ, IL-5, IL-10, IL-13 and IL-33 in all studied groups. Data represented as mean ± SEM (n = 7), values with different superscript letters are considered statistically significant (*P* < 0.05).

**Figure 8:**
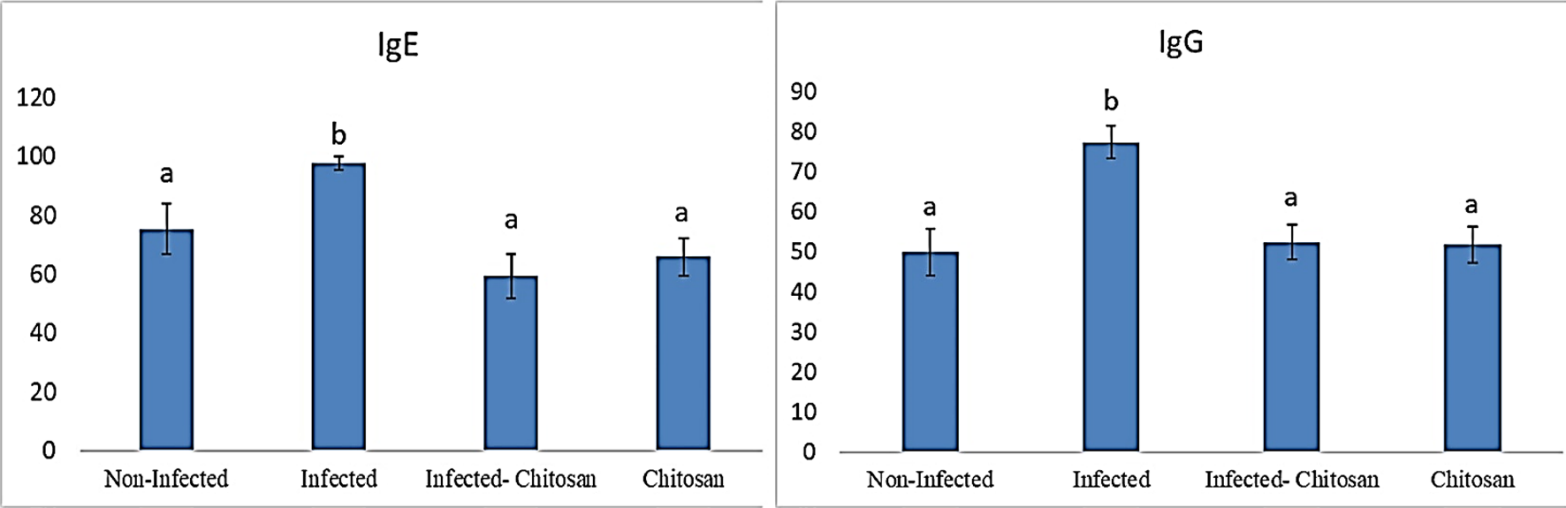
Concentrations of total IgE and IgG in all studied groups. Data represented as mean ± SEM (n = 7), values with different superscript letters are considered statistically significant (*P* < 0.05).

Indirect ELISA was used to measure humoral responses to a natural *S. muris* infection in laboratory rats; levels of IFN- **γ**, IL-5, IL-13, and IL-33 were considerably (*P* < 0.05) higher in infected rats than the non-infected ones. At the same time, IL-10 showed no significant variation in any of the groups examined (Fig. 7). Additionally, it was found that the total IgE and IgG concentrations (Fig. 8) of infected rats were significantly higher (*P* < 0.05) than those of the control group; however, chitosan treatment returned these elevations.

### Histopathology

**Figure 9:**
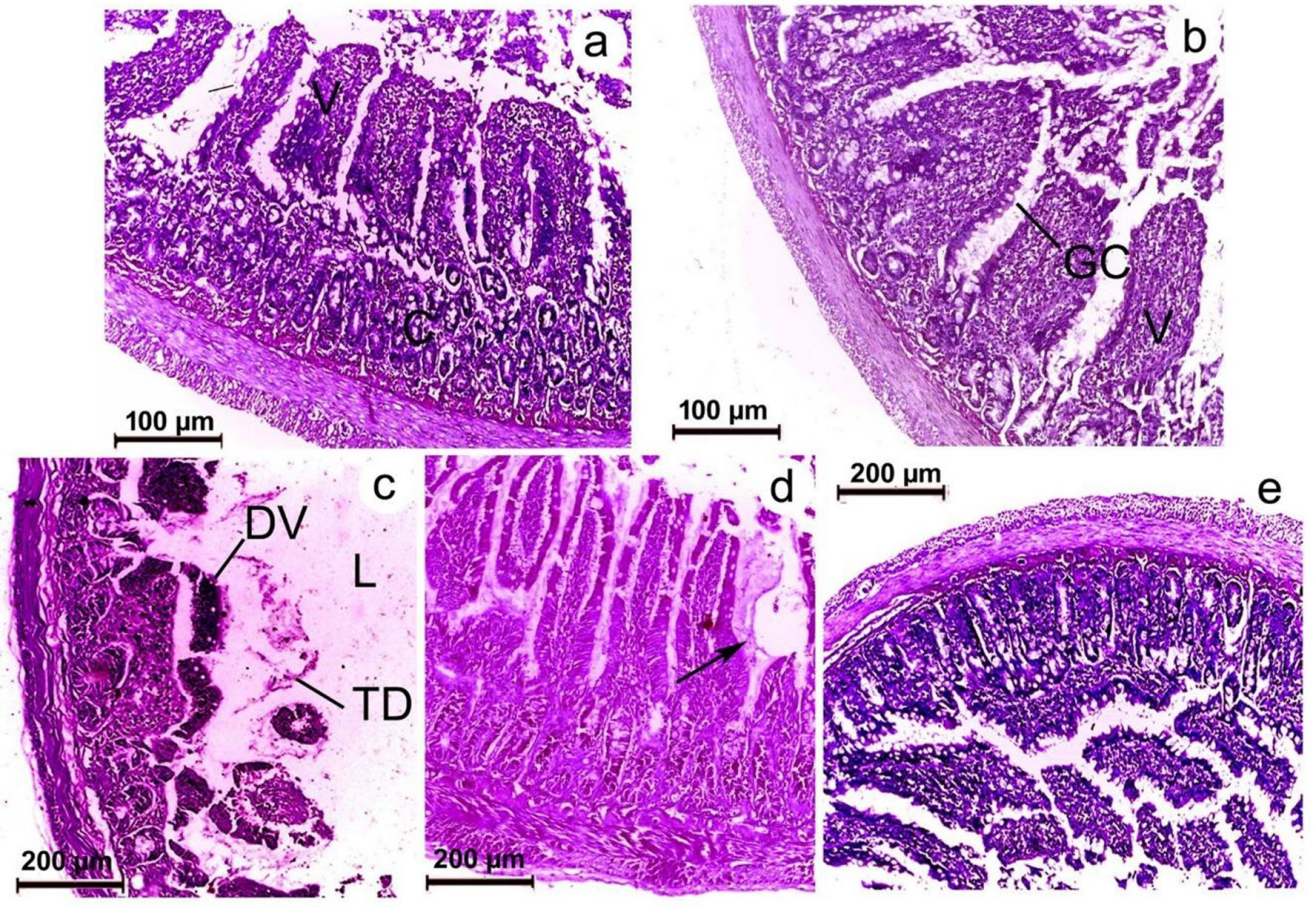
Photomicrographs of rat colon stained with hematoxylin–eosin. (**a**) Control group showing regular arrangement of different intestinal layers and intact finger-like villi. (**b**) Non-infected (received chitosan) showing typical arrangement of intestinal layers with normal villi (**c**) Infected group showing epithelial damages, altered mucosal architecture, deformed villi (DV) and tissue debris (TD) (**d**) infected rats showing worm-burrowing villi (arrow) (**e**) chitosan-treated group displaying well defined intestinal architecture.

Intestinal sections (colon region) of control and chitosan (non-infected) groups exhibited normal appearance of all layers. In contrast, the infected group displayed certain pathological abnormalities, including erosion and villi destruction. The parasites that deeply penetrated the mucosa also disturbed and destroyed the muscularis. On the other hand, the chitosan-treated group showed typical architecture as the control one (Fig. 9).

**Figure 10:**
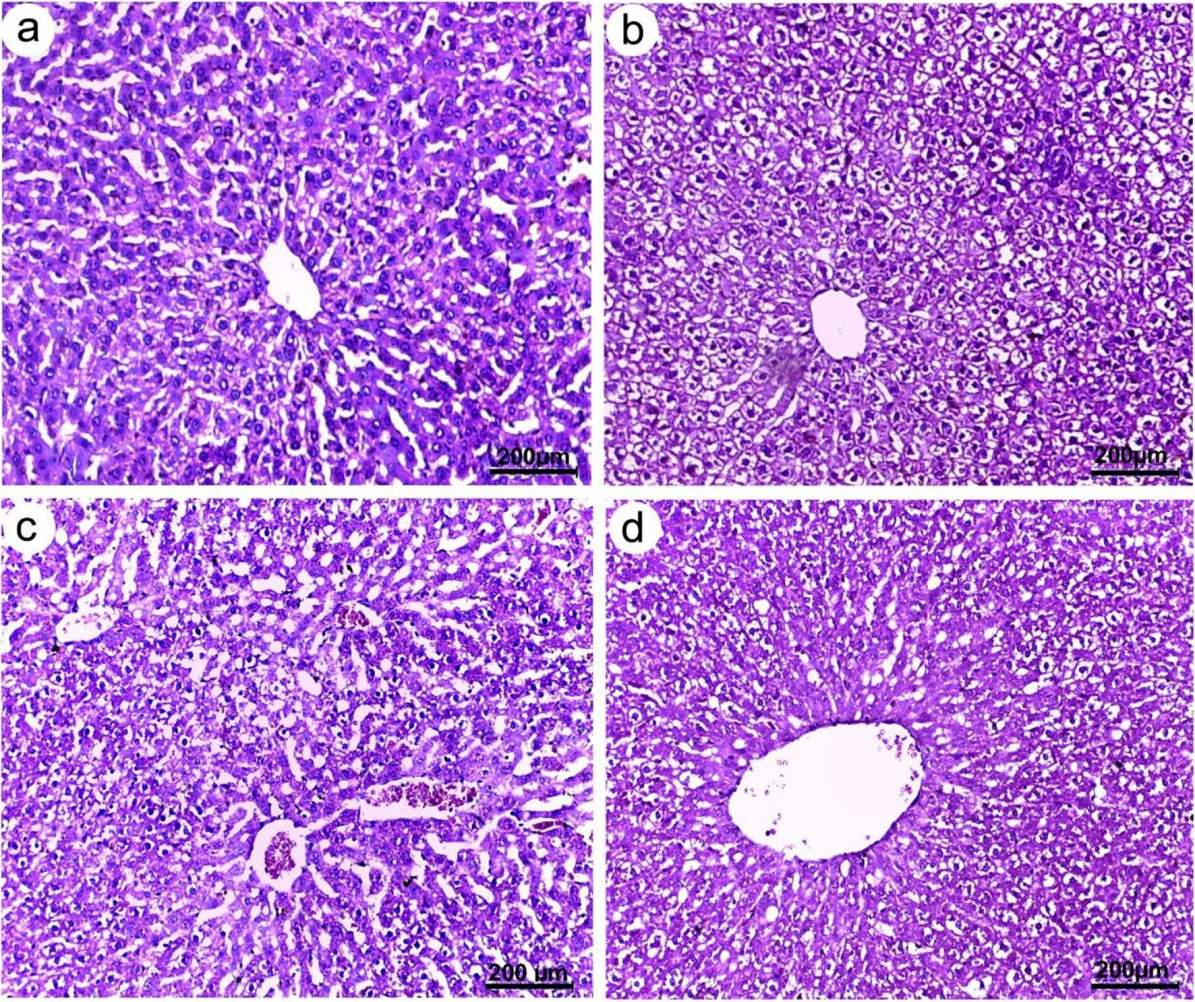
Photomicrographs of liver sections of Wistar rat (**a**) control (**b**) chitosan (non-infected) group (**c**) infected group (**d**) chitosan-treated group, all showing normal hepatic architecture with polygonal hepatocytes and central vein displaying relatively normal appearance.

**Figure 11:**
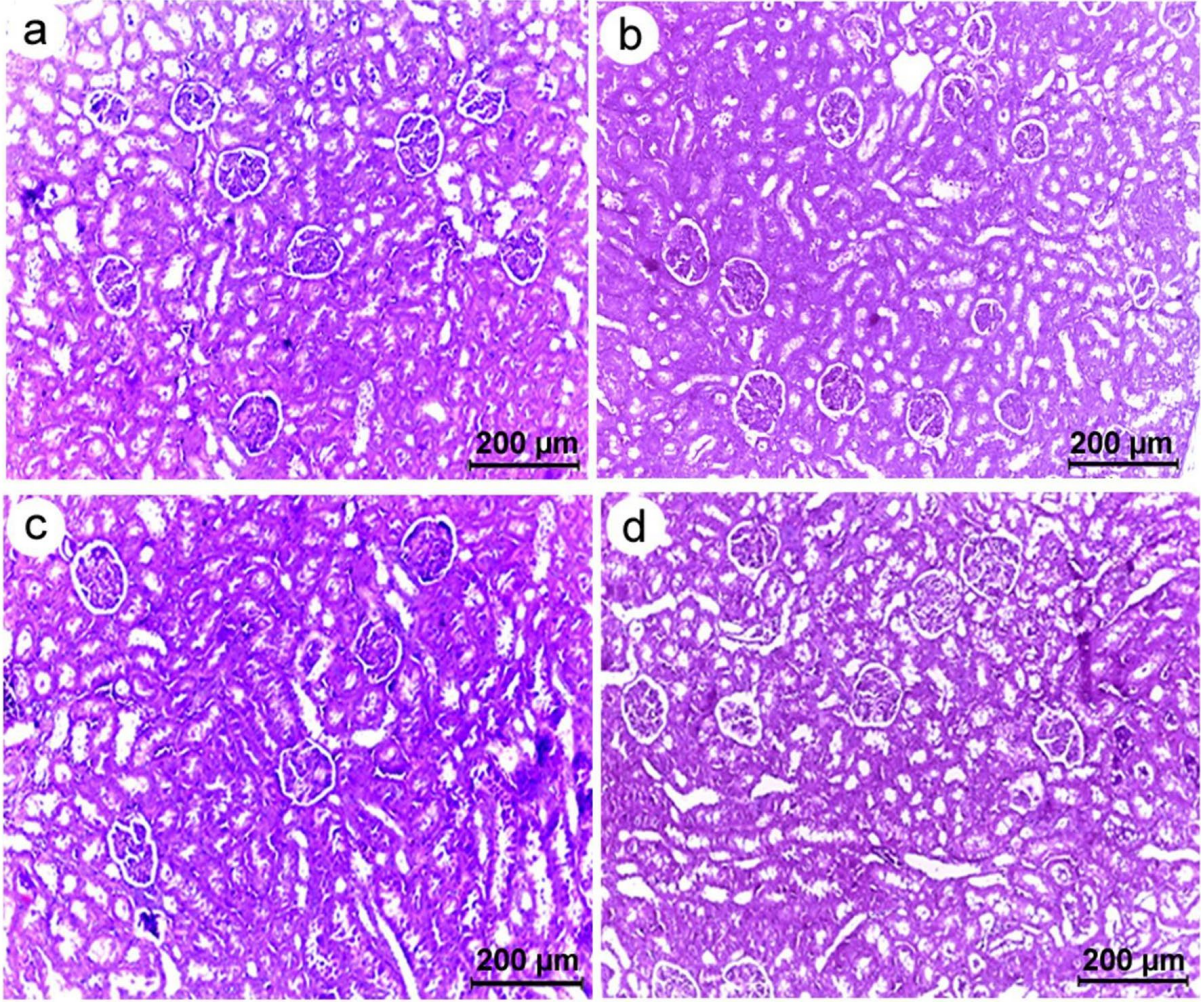
Photomicrographs of kidney sections of Wistar rat. (**a**) Control (**b**) chitosan (non-infected) group (**c**) infected group (**d**) chitosan-treated group, all showing regular renal architecture with typical appearance of glomerulus and convoluted tubules.

**Figure 12:**
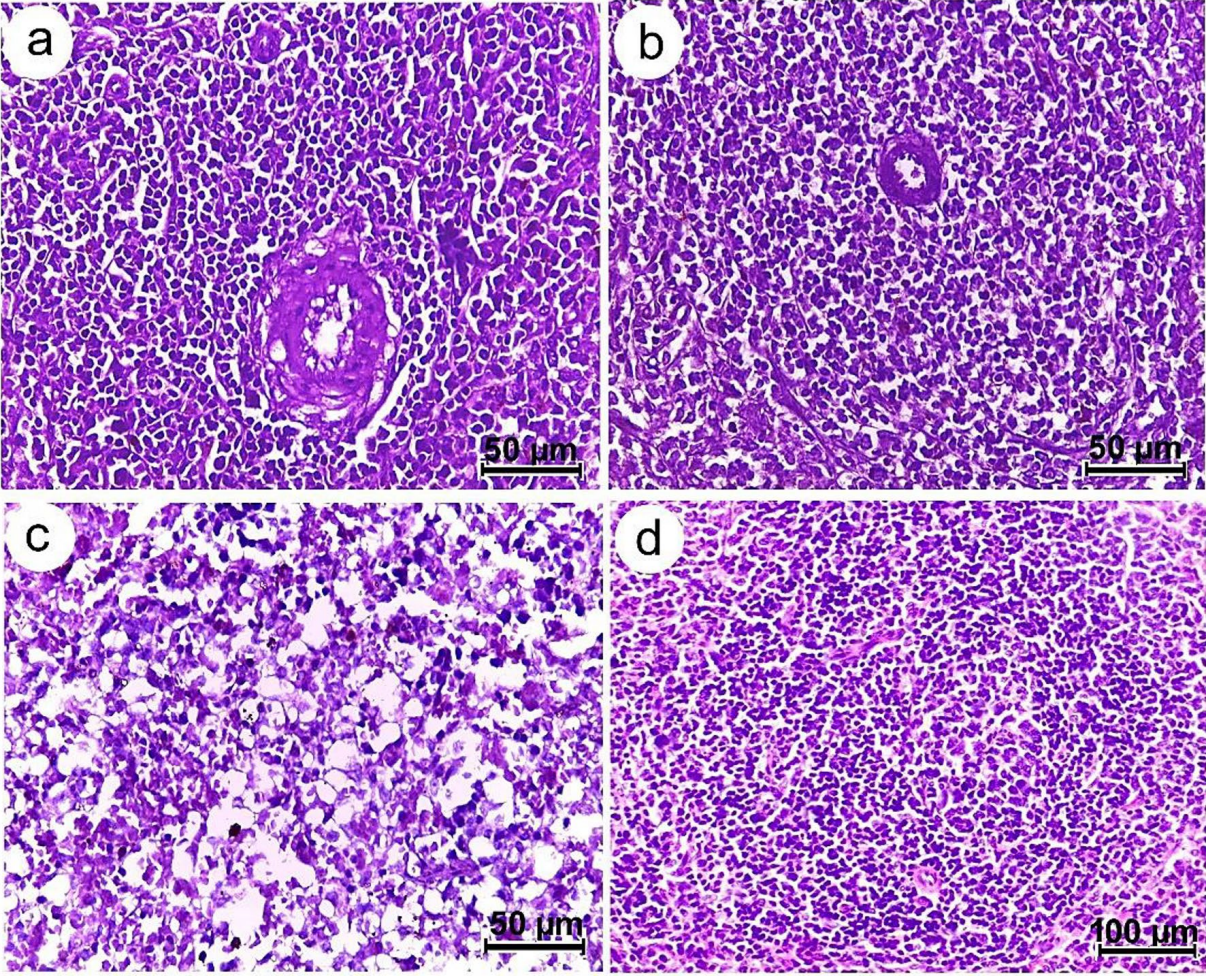
Photomicrographs of splenic sections of Wistar rat (**a**) control (**b**) chitosan (non-infected) group showing well defined splenic architecture including healthy lymphoid cells, sinuses and central artery (**c**) infected group showing deposition of hemosiderin pigment in the splenic parenchyma as well as lymphoid depletion (**d**) chitosan-treated group displaying normal cellular appearance.

Liver sections of all studied groups showed normal appearance with well-organized polygonal hepatocytes, homogeneous cytoplasm, and sinusoids dispersed randomly throughout the hepatocytes (Fig. 10). The distribution of glomeruli and tubules in the renal tissues also appeared normal (Fig. 11). Furthermore, the lymphoid follicles in the spleen sections from the control and chitosan (non-infected) groups were well-structured (Fig. 12a,b), while those from infected rats displayed histopathological changes that were accompanied by lymphoid depletion and deposition of hemosiderin pigment in the splenic parenchyma (Fig. 12c). In contrast, following chitosan treatment, spleen tissue restored its normal appearance (Fig. 12d).

## Discussion

The unique physical and chemical properties of chitosan make it valuable in various biomedical applications^29^. In the current study, FTIR spectroscopy of chitosan particles revealed peaks corresponding to their functional groups, which agrees with other published studies^30–33^. Results demonstrated that chitosan treatment caused a reduction in the adult count of *S. muris* by 87.5% in treated rats compared to the untreated group. These findings are consistent with those of Abdel-Latif et al.^34^, who reported that chitosan particles reduced the adult and egg counts of *Hymenolepis nana* infecting mice. Also, it was found that chitosan particles caused the deformation of the cephalic region and body cuticle of the recovered worms. Similarly, Salem et al.^35^ reported that treatment with chitosan nanoparticles led to the disintegration of lips and rupture of the cuticle of *Ascaridia columbae*, infecting pigeons. Another parallel study by Abu-Elala et al.^36^ confirmed that chitosan-silver nanocomposite potently controlled *Lernaea cyprinacea* infection in goldfish aquaria. Currently, the nematicidal mechanisms of chitosan and its derivatives are unknown; however, Badawy and Rabea^37^ hypothesized that when positively charged chitosan molecules (charge on C-2 of the glucosamine monomer) interact with negatively charged microbial cell membranes, proteinaceous and other intracellular contents leak out.

Infected rats revealed an elevation in AST, ALT, ALP urea, and creatinine levels. On the same line, Rahimi et al.^38^ and Kot et al.^39^, reported that the abnormalities in liver and kidney functions can be caused by parasitic diseases, resulting in various biochemical changes in the host body. Conversely, liver and kidney parameters were within the normal range and did not differ significantly between the control and chitosan-treated groups, indicating normal kidney and liver functions. These findings were supported by Ugbaja et al.^40^, who found that chitosan improved hepatic and renal biomarkers against hyperlipidemia-invoked damages in Wistar rats.

Parasitic infection may also elicit oxidative stress, leading to an increase in reactive oxygen species (ROS) formation in tissues and a decrease in antioxidants, which damages membranes, DNA, and protein structures^41^. Data revealed that infection with *S. muris* caused a significant drop (*P* < 0.05) in GSH content and SOD and CAT activities, as well as an increase (*P* < 0.05) in both MDA and NO concentrations compared to the control rats. In line with these findings, Ince et al.^42^, found that *S. muris* induced oxidative stress in rat liver tissue by increasing the level of MDA while decreasing GSH, SOD, and CAT levels. Similarly, Da Silva et al.^43^ found that *T. evansi* infection in camels resulted in inhibition of the antioxidants GSH and SOD. Also, Saleh et al.^44^ reported that camels naturally infected with *T. evansi* revealed a decline in SOD activity. These antioxidants may be consumed as free radical scavengers during the oxidative process in the natural infection^44^^.^ On the other hand, following chitosan treatment, these alterations reversed to their normal state like control group. Ozdek et al.^45^ also reported that chitosan showed a protective effect on the kidneys against lead-induced oxidative stress in rats by decreasing MDA concentration and increasing CAT activity. Nomier et al.^30^ found that chitosan nanoparticles provided substantial protection and amelioration against CCl4-induced oxidative stress by decreasing malondialdehyde levels and increasing the depleted reduced glutathione levels. Mechanisms related to the antioxidant activity of chitosan include free-radical scavenging action, metal-ion-chelating ability, and reducing activity^46,47^.

Helminth infections are linked with sophisticated immunomodulatory mechanisms that impact all aspects of the host immune response to maintain survival^48^. Intestinal nematodes can induce Th2 immune responses via the production of cytokines IL-4, IL-5, IL-13, and IL-33, as well as elevated immunoglobulin IgE, which are crucial for the control of infections^49,50^. Many publications mainly cover the immunological responses caused by *Syphacia* spp., but little discussed the immune response profile after medication^51,52^. This study demonstrated that *S*. *muris* promoted the production of cytokines levels of IFN- **γ**, IL-5, IL-13, and IL-33, which were considerably (*P* < 0.05) higher in infected rats than the control ones. At the same time, IL-10 showed no significant variation in any of the groups examined. Similarly, Michels et al.^53^ reported that *S. obvelata* infection resulted in elevated levels of IL-5 and IL-13. Also, Humphreys et al.^54^ and Neill et al.^55^ observed that nematode infection led to a rise in IL-33 levels. In a similar vein, Shlash et al.^56^ found higher levels of IFN- γ, IL-5, and IL-13 in serum samples from *A. duodenale*-infected patients. On the other hand, after chitosan treatment, a significant decline in IFN- γ, IL-5, and IL-13 levels was observed due to a decrease in worm burden, which may reduce the immunological compression and allow the cytokines to revert to their resting state as in the control group. This result agreed with Abdel-Latif et al.^34^ who reported the anti-inflammatory action of chitosan particles by downregulating IFN- γ in the infected rats.

Immunoglobulins IgE and IgG detection is essential for diagnosing numerous pathological conditions associated with bacterial, viral, and parasitic infections, thereby enabling the administration of the appropriate therapeutic treatment^57^. Our data revealed a significant rise (*P* < 0.05) in total IgE and IgG levels in infected rats compared to the control group, which agrees with Michels et al.^52^ and Perec and Okulewicz^57^, who demonstrated an elevation in *Syphacia*-specific antibodies. Also, Wright and Bickle^58^ stated an increase in hookworm-specific IgG and IgE levels in humans following infection. Herein an increase in total IgG level in infected rats may be associated with the production of INF- γ a Th1 product as suggested by Binder et al.^59^ and Cetre et al.^60^. The remarkable rise in IgE level in infected rats in the current study may be associated with IL-13 production rather than the tested IL-10 which showed no significant change. This finding is consistent with Taghipour et al.^49^ who reported that Interleukins, IL-4, IL-10 and IL-13 are primarily responsible for IgE production by inducing B-cell switching. Interestingly, after treatment with chitosan, total IgE and IgG concentrations returned to normal levels. As postulated by Jiang et al.^61^, chitosan administration lowered serum IgE and IgG1 levels as well as Th2 cytokine levels (IL-4, IL-5, and IL-13) in a mouse model.

Histopathological investigations displayed that *S. muris* caused some alterations, including atrophy and distortion of villi, resulting in the loss of the normal appearance of the intestinal layers. On the same line, Plachý et al.^1^ found that a high pinworm burden caused histological changes in intestinal layers. Also, Anwar et al.^62^ detected degenerations of the intestinal mucosa and atrophy of the villi of the terrestrial rodent *Psammomys obesus* due to helminthic infection. Saracino et al.^63^ observed a marked structural alteration of the mucosal layer of rats infected with *Trichinella spiralis*. In contrast, intestinal sections of chitosan-treated rats exhibited normal architecture with a regular appearance of villi. This finding is supported by Abdel-Latif et al.^34^, who demonstrated an improvement in intestinal tissues of mice infected with *Hymenolepis nana* following chitosan treatment.

The liver and kidney sections of all studied groups displayed a normal appearance, however, the splenic tissues of the infected rats showed lymphoid depletion. On a similar vein, John^64^ documented abnormalities in splenic lymphoid tissue related to nematode infection. Thus, the recorded pathological changes in splenic tissues may be related to *S. muris* infection, which also induces detrimental effects on host physiology, including oxidative stress. Fahmy and Diab^65^, also reported that tissue damage and inflammations in the histopathological sections of infected rats was attributed to the increased levels of reactive oxygen species (ROS). On the other hand, it was found that chitosan improved the histopathological alterations in spleen. Similarly, Abd El-Fattah et al.^66^ demonstrated that chitosan alleviated most of dioxin's biochemical and histological effects due to its antioxidative properties. Also, Wasso et al.^21^ stated that there are no pathological changes in the main organs in goats infected with gastrointestinal strongyles treated with chitosan-encapsulated bromelain. These findings in line with Li et al.^67^, who reported that chitosan is a non-toxic, biodegradable, and biocompatible natural product with numerous medical applications in preventing or treating infectious diseases.

## Conclusions

Our study showed a potential anthelminthic effect of chitosan particles against *S. muris* infecting laboratory rats by reducing the worm burden, decreasing oxidative stress, and recovering the pathological alterations of host tissues. Interestingly, this is the first study to investigate the immune-protective effect of chitosan against *S. muris* infection, referring to its anti-inflammatory properties by downregulating IFN-γ, IL-5, IL-13, IL-33, IgG and IgE. Therefore, chitosan can act as good alternative to synthetic drugs where anthelmintic resistance has developed. However, further studies should evaluate the activity of acetylcholinesterase (AChE) in vitro in the presence and absence of chitosan particles to detect its impact on the nervous system of gastrointestinal nematodes (GI).

## Data Availability

The data analyzed are saved during the current study and available from the corresponding author on request.
